# Supplementary material for: Are carbon emissions trading and green financial instruments synergistic? -Comprehensive quantitative research based on content analysis
Source: PLoS One. 2024 Mar 7;19(3):e0298601. doi: 10.1371/journal.pone.0298601 (PMC10919601; doi:10.1371/journal.pone.0298601)
Supplement: S1 Data — (ZIP) [file pone.0298601.s001.zip › Data/地级市Z_F工作报告.zip!/╣π╢1⁄2/├»├√╩╨/└┤╫╘╣1⁄2╓┌║┼í╛┬φ┐╦╩2╛▌═°í┐_2014.docx]

各位代表：

我代表市人民政府向大会作工作报告，请予审议，并请政协各位委员和其他列席人员提出意见。

一、2013年工作回顾

过去一年，在省委、省政府和市委的正确领导下，在市人大及其常委会和市政协的监督支持下，市政府紧紧围绕2015年底人均生产总值达到全国平均水平的总目标，抢抓粤东西北地区振兴发展的重大机遇，经济运行提速增效，社会建设稳中向好。全年全市完成生产总值2160.2亿元，增长13.2%，同比提高2.6个百分点，增速居全省第五；人均生产总值达36063元，增长11.9%。

一年来，我们做的主要工作及成效如下：

（一）狠抓第一要务，经济发展快中见好。

紧紧扭住加快发展这一中心任务，凝心聚力，积极作为，经济保持持续快速健康发展良好势头。

发展速度持续加快。经济增长逐季提速，在2012年增长10.6%基础上，去年一季度增长11.6%，上半年增长12.6%，前三季度增长13.1%，全年增长13.2%。主要经济指标增速在全省排位靠前，3项指标居全省第一，2项指标居全省第二。

发展质量有效提升。公共财政预算收入90.3亿元，增长15.6%，非税比重控制在39.6%，同比下降4个百分点。新增规模以上工业企业218家,工业增加值529.1亿元，增长17.1%。全市规模以上工业企业实现利润总额111.3亿元，增长24%。城镇居民可支配收入20036元，增长11.1%；农村居民人均纯收入10704元，增长12.6%。

发展动力不断增强。全市完成固定资产投资660.5亿元，增长54.6%，增速连续两年居全省第一，总量排名比上年上升三位，在粤东西北地区排第四位；工业投资383.8亿元，增长75.9%；民间投资474.1亿元，增长79.1%。进出口总额12.2亿美元，实际利用外资1.1亿美元，分别增长17.7%和43.3%。人民币贷款余额637.1亿元，增长18.1%。

（二）加强基础设施建设，发展环境逐步优化。

以交通大会战为重点，推动全市基础设施建设大发展，全年共完成交通基建投资42.7亿元，增长132.9%。

对外交通建设取得突破性进展。包茂高速茂名段征地拆迁工作基本完成，便道全部拉通，路基、桥涵、隧道进展较快，完成投资占年度计划136.3%，创造了我省高速公路建设的“包茂速度”。汕湛高速云浮至湛江段已确定投资主体。茂湛铁路茂名段建成通车。深茂铁路茂名段正进行全线初步设计定测。博贺疏港铁路已成立联合筹备组。湛江机场迁建确定首选场址。

对内交通投资力度加大。环城东路二期、西城西路、悦民路北段建成通车。新港大道南段10.8公里完成路面主体工程，尾段2.7公里已动工建设；新港大道北段完成6公里基层、3公里混凝土路面施工。海洋大道东段、包茂高速茂名东连接线（油城十路）正加紧施工。茂化公路S372线首期工程、山根公路改造项目首期工程已动工建设。S282、S284、S370线道路改造工程共完成投资3380万元，S280线高长二期改造工程开工建设，G207线信宜至高州段路面改造工程已获省发改委批复立项，G325线茂名段改造工程争取补助资金1.27亿元。

环保基础设施明显改善。河西城区生活污水处理厂全面开工建设，白云江高（电白）、东莞大朗（信宜）产业转移园污水处理厂建成投用，全市新增污水处理能力1万吨，新增建设管网65.6公里。大力实施“城乡清洁工程”，投入资金5325万元加强环卫设施建设。市生活垃圾焚烧发电项目、高州市垃圾填埋场建成投用，化州市、滨海新区（电白）垃圾填埋场基本建成，信宜市垃圾填埋场完成土建工程，106个镇（街）建成垃圾转运（压缩）站。

水利电力设施改造加快。全市水利建设完成投资6.3亿元，高州水库灌区续建配套与节水改造首期工程完成渠道改造139.6公里，高州水库恢复89.0米正常蓄水位淹没处理项目完成立项。电网固定资产建设完成投资7.5亿元，进一步优化主网架构，那务输变电工程建成投用，消除茂名北部地区大面积停电风险；加强配网改造，农村居民端电压合格率达97.9%，同比提升7.9%。

（三）做实发展平台，滨海发展格局初步显现。

创新体制机制，启动博贺湾海洋经济综合试验区和水东湾新城建设，与高新区一起构成新区发展三大实体平台。经过近一年的努力，三大平台发展迅速拉开了架势、打开了局面。

博贺湾经济区呈现大开发场景。东阁岭征地全面完成，签订征地协议共计6000多亩。粤电煤炭码头项目防波堤已推进900多米，粤电煤炭发电项目正加快场地平整工作。博贺新港区东西防波堤、博贺湾大道开工建设。

高新区开发空间得到极大拓展。西南片区全面启动建设，工业大道和高新大道等路网加快施工。乙烯厂卫生防护距离搬迁安置工作基本完成。全年共征地兑款2245亩。新建成项目20个，累计建成和在建项目86个，其中顺丁橡胶项目建成投产，巴斯夫异壬醇项目开工建设，聚丙烯项目进展顺利。高新区获评“2013年中国化工园区20强”。

水东湾新城重大基础设施建设逐步启动。水东湾综合整治项目全面推进，渔排网箱清理已签订拆除协议99%，拆除56.1%。茂名职业技术学院新校区项目开工建设。新城重点项目签订征地协议1386亩。投资54.89亿元的水东湾综合整治开发项目完成招标，近期将动工建设。

（四）坚持统筹发展，城乡面貌加快改善。

城市环境进一步优化。完成了光华路升级改造，建成了官渡四路。油城一二三路改造和西粤北路建设项目正抓紧施工。油城七八九路和人民南、北路完成沥青罩面。启动矿业北路改造工程。新增城区绿地面积约5万平方米，人均公园绿地面积达12平方米。摩托车治理取得良好效果。支持广东石油化工学院校区扩建，完成400多亩征地。全市新增绿道7.5公里。

县域发展进一步加快。县域工业园承载能力大幅提升，新增用地储备3615亩。信宜人民公园、锦江桥、碧水湾大桥，高州“七路一桥一中心”，化州“三江六岸”和“三大出口”，电白“七路一桥一广场”等道路市政设施加快推进。6个县（市、区）主要经济指标增速高于全市平均水平，其中规模以上工业增加值增长28.1%，固定资产投资增长62.2 %，公共财政预算收入增长21.5%，县域经济成为拉动全市经济发展重要力量。

农业农村稳定发展。农业在大灾之年保持平稳增长。加强农产品市场营销体系建设，大宗农产品收购价普遍比上年提高10%以上。农业产业化水平明显提升，新增省级农业龙头企业8家，农民专业合作社发展到3051家，总量位居全省第一。农产品质量安全保持稳定，连续7次全省抽检合格率100%。新增省级名牌产品12个。创建了一批宜居示范城镇、村庄。全省首个动物无害化处理中心和政策性育肥猪保险在高州启动。

（五）深入推进改革开放，发展活力有效激发。

体制改革进一步深化。行政审批制度改革扎实推进，大幅压减行政审批事项，市一级共取消271项，下放和委托119项，调整比例达45.8%。深入推进简政强镇事权改革，规范设置镇级机构，大幅向镇级下放事权，强化了镇级社会管理、公共服务职能。全市综合政务服务体系不断完善，109个镇（街）行政服务中心均建成投用。市县镇三级联动网上办事大厅全面建成。组建统一的市公共资源交易中心。顺利推进市卫生、计生和食品药品监督管理体制改革。进一步完善经济功能区体制机制，充分释放经济区发展活力。国企改革深入推进，组建了茂名港集团、交投集团、文化传媒集团。理顺市辖区土地征收、储备和经营机制，推动形成规范统一的土地市场。

开放水平进一步提升。出台《茂名市招商引资管理及奖励暂行办法》，去年全市招商引资竣工、新开工项目累计539个，总投资额601.8亿元。与中海油气电集团签订粤西LNG项目合作协议，引进了中交集团、太平洋建设集团等一批大型企业。资本市场发展取得突破，交投集团发行10亿元企业债券获批，茂名港集团完成8亿元中票发行前期准备工作，东亚银行在我市设立分支机构。

地企合作迈上新台阶。与茂名石化公司建立了主要领导和分管领导联席会议制度，完成了露天矿区整体移交，合资组建了茂名石化南海精细化工有限公司。与省国资委和省属企业加强对接，与粤电集团合作进一步深化，茂名热电厂7号机组获国家发改委核准。

（六）努力保障和改善民生，各项社会事业扎实推进。

民生实事取得新进展。全年民生支出164.5亿元，占全市公共预算支出75.3%。培训农村劳动力4万人，实现转移就业8万人，实现城镇新增就业7.5万人，城镇登记失业率2.36%。全市养老机构增加床位2538张，增长26.9%。新开工建设保障性住房2303套，基本建成2453套。新一轮扶贫开发“双到”工作进展顺利，投入资金6.2亿元，启动对村帮扶项目2307个。完成农村低收入住房困难户住房改造9455户，完成“两不具备”村庄搬迁3037户。积极推进灾后复产重建，全市2784户“全倒户”春节前入住新居。

社会保障水平明显提升。推动社保惠民，企业职工养老保险、城乡居民养老保险和基本医疗保险参保率分别达81.2%、99%和98.5%。低保五保救助水平大幅提高，低保城镇和农村月人均补差分别比上年提高116元和35元，五保集中供养和分散供养年人均水平分别比上年提高3259元和3012元。在全省率先建立孤儿基本生活养育标准自然增长机制。城乡居民医保筹资标准提高到每人每年330元，住院支付比例提高5至15个百分点。全面实现医保“一站式”结算。

社会事业取得新成绩。投入18.6亿元推动教育创强，51个创强镇(街)已全部向省申报督导验收。整合中等职业教育资源，电白职校与市一职基本完成整合，电白职校新校区已移交茂名卫生学校。科技工作取得新成绩，获得省科学技术奖6项，居粤东西北地区前列；全市专利申请2530件，专利申请总量居粤西首位。公共文化服务体系不断完善，文化惠民成效明显。深化医药卫生体制改革，全面推进区域医疗卫生信息化和镇村医疗一体化建设。突出抓好传统节假日体育活动、体育节、南粤幸福周活动和体育“三下乡”，群众体育活动蓬勃开展。食品药品监管进一步加强。着力构建立体化治安防控体系，严厉打击违法犯罪，社会治安大局总体平稳。坚决遏制重特大生产安全事故，全市安全生产形势平稳。高度重视和加强人口计生工作，全力稳定低生育水平。民族宗教、统计、物价、国防动员、人防、双拥、气象、地震、残联、红十字、外事、侨务、档案、方志等工作取得新的成效。

同时，政府自身建设不断加强。坚持向人大及其常委会报告工作，向人民政协通报情况，自觉接受监督，办理人大代表建议101件、政协委员提案274件。加强行政复议和行政应诉工作，落实行政执法责任制。加强行政监察、审计监督和政务公开。开展专项效能监察，加大问责力度，全年共问责180人。加大民主评议政风行风和纠风工作力度，政府执行力明显提升。

去年政府工作报告决定的116项工作，有109项按期完成，有7项正在稳步推进。对没能按时完成的事项，我们将切实加大推进力度，逐项跟踪落实。

各位代表！过去一年，我市取得的成绩来之不易。这是在省委、省政府和市委的正确领导下，全市人民齐心协力、奋力拼搏的结果。在此，我代表市政府向全市人民，向驻茂人民解放军、武警官兵和各民主党派、人民团体、社会各界人士致以崇高敬意！向所有关心支持茂名发展的港澳同胞、台湾同胞、海外侨胞及国际友人，表示衷心感谢！

我们也清醒地认识到，我市加快发展面临的交通、土地、资金、人才瓶颈制约仍然突出；地方工业仍然弱小，产业结构单一；地方财力薄弱且收入质量不高；营商环境和执行力还有较大提升空间；城镇化水平不高，中心城区集聚力、辐射力不强，城市管理水平不高；政府职能有待进一步转变；机关作风建设有待进一步加强。对此，我们将高度重视，认真解决。

二、2014年工作安排

各位代表！2014年是全面深化改革的第一年，是完成“十二五”规划目标任务的关键一年。当前，我市经济发展呈现良好态势，无论是产业、税收还是投资，都有不少增长点，今年拟投产重点产业项目新增产值近300亿元。同时，我市已进入大开发、大建设时期，包茂高速和深茂铁路全面建设，博贺湾经济区、高新区西南片区、市民片区、水东湾新城等多个区域进入投资高峰期，各县（市）城区扩容提质和工业园区建设全面推进，这些有利因素将为全市经济较快发展提供有力支撑。

2014年政府工作的总体要求是：全面贯彻落实党的十八届三中全会、省委十一届三次全会和全省进一步促进粤东西北地区振兴发展工作会议精神，按照市委十届四次全会的部署要求，紧紧围绕2015年底人均生产总值达到全国平均水平的总目标，以发展为中心，以改革为动力，加快完善交通基础设施，推进产业园区建设，力促城市扩容提质，办好民生实事，推动茂名振兴发展。

今年经济社会发展主要预期目标是：生产总值增长12%，规模以上工业增加值增长20%，固定资产投资增长30%，社会消费品零售总额增长12%，外贸进出口总额增长7%，公共财政预算收入增长13%，居民消费价格总水平涨幅控制在3.5%左右；城乡居民人均收入实际增长与经济增长同步，节能减排约束性指标完成省下达年度计划。

为完成今年目标任务，并在实际工作中力求完成得更好一些，我们务必抢抓发展机遇，集中精力资源，突出抓好六个方面工作：

（一）着力加快交通建设，改善区位条件。

推进对外交通高速化。尽快完成包茂高速茂名段征地拆迁收尾工作，路基、桥涵全部完成，路面全面开工。加快推进汕湛高速茂名段、湛江机场迁建和茂名粤西空港物流园前期工作。开工建设深茂铁路茂名段，征拆工作要在沿线各市中率先完成。配合做好广茂铁路、河茂铁路电气化改造。加快洛湛铁路茂名段沿线站场及进站公路建设，加强沟通协作，提升客货运量。加强物流站场规划建设，加快推进茂名综合客运枢纽和电白技术作业编组站物流中心前期工作。

推进对内交通快速化。加快推进茂化公路、山根公路建设。开工建设G207信宜至高州段路面改造、G325线茂名段路面修复工程。完成包茂高速茂名东连接线（油城十路）、新港大道北段和海洋大道东段建设，力争完成新港大道南段建设。加快开展市民大道（二期）工程、包茂高速茂名东连接线余下6.7公里前期工作，力争尽快开工建设。开通沈海高速（广湛）鳌头出口。加快推进危桥改造，完成不少于500公里的农村公路硬底化建设任务。

推进港口建设现代化。做好《茂名港总体规划（修编）》报批工作。加快推进粤电煤炭码头和博贺新港区东西防波堤工程。加快广州港、中化码头前期工作，年内开工建设广州港码头项目。加快推进博贺新港区30万吨级（首期15万吨级）航道工程报批工作。力争年内开工建设水东港区3万吨级航道工程。加快推进博贺疏港铁路项目前期工作。着手谋划博贺新港区口岸配套设施建设。

（二）着力加快园区建设，力促实业兴茂。

加快博贺湾经济区开发建设。加快博贺湾大道建设。加快推进博贺湾大桥、滨海大道等项目前期工作。完成吉达港区石化工业园区1平方公里、博贺临港工业园区2平方公里基础设施和浪漫海岸二期建设，开工建设广东圣浩天然纤维素一体化项目和东达金海岸旅游开发项目。加快粤电煤炭发电项目、粤西LNG项目各项前期工作，开工建设LNG项目陆域形成工程。

继续做大做强高新区。围绕建立国家级高新区目标，按“整体策划，重点推进”思路，加快西南片区开发，突出完善道路网络主骨架，同步建设管廊管线、污水处理、水电消防等重要设施，争取建设国家级安全生产应急救援基地。尽快启动芳烃项目，全年力争新开工项目20个。保护好露天矿采矿场和南北排土场林木及矿产资源，加快河西老工业区整体改造。

加快推进县域工业园区扩能增效。推动产业向园区集中，园区向城市集中。创新园区投资建设运营模式。探索以“一园多区”等形式推动园区整合。抓好骨干企业培育和发展，支持现有企业增资扩产。支持高州、化州、茂南、茂港、博贺湾经济区申报纳入省产业转移工业园管理。加强园区用地保障，全市土地年度利用计划指标不少于15%用于园区项目建设；厉行节约集约用地，设立提高园区投资门槛。

同时，加快现代农业发展步伐。稳定粮食生产，力争全年粮食总产145万吨以上。积极引进和推广新品种，发展名优特色种植业。大力推动南药产业发展。抓好现代高标准农田和中低产田改造建设，提高农业综合生产能力，完成高标准基本农田建设37.95万亩。推进农业产业化，大力发展农业龙头企业、农民专业合作社、家庭农场等新型经济组织。培育畜牧业名优产品。抓好农产品宣传推介，组织好大宗农产品产销对接会。依托电子商务和物流业发展现代农业，力促茂名特色水果上线交易。推进深海网箱养殖和远洋捕捞，巩固渔业大市地位。

（三）着力推进城市扩容提质，加快城镇化进程。

起步建设水东湾新城。水东湾新城是茂名未来城市的核心所在。全力做好撤销茂港区、电白县，合并设立电白区各项工作。完成水东湾渔排网箱清理，建设美丽海湾。尽快组建水东湾新城控股集团，启动水东湾综合整治开发项目，动工建设水东湾大桥、海洋公园（一期）。推进市人民医院新城院区项目，年内开工建设。确保职业技术学院新校区项目9月部分建成投入使用。

同时，加快推进市民片区建设。进一步完善路网，加快市民活动中心、市民公园后续项目和颐年苑等项目建设，大力支持粮油综合批发市场等项目建设。适度推进配套商住项目开发，聚集人气，带旺物流。

加强北组团城市建设管理。进一步完善东北五小区路网，年内动工建设官渡三路、高凉北路（官山路至官渡路段）和文明北路（官渡路至大园路段），加快推进西粤北路建设。完成矿业北路、油城一二三路改造工程。加快高山桥改造工程建设，推进官渡一二路、站前三四路和天桥路沥青罩面改造。切实推进市区水浸黑点整治改造工作。调整增加停车场、农贸市场、垃圾压缩站、公厕等城市配套设施。着手启动茂南新城（站南片区）开发建设。加强城市管理，坚决制止和查处违法建设，整治乱摆乱卖乱停现象。规范户外广告经营管理。

大力推进各县（市）中心城区扩容提质。认真落实国家新型城镇化规划和我省实施意见，坚持遵循规律、因势利导推进城镇化。加快各县（市）城区基础设施建设，推进新片区开发，提升中心城区承载力。提高城市公共服务水平，突出抓好住房、医疗、教育等工作，促进外出务工人员回流。引导商品房增加中小户型，满足城镇居民多样化需求。推进城乡建设用地增减挂钩试点，优化国土资源开发利用布局。加强宜居城乡建设，深入实施“城乡清洁工程”。

（四）着力推动改革开放，增创发展新优势。

深化行政管理体制改革。深入推进行政审批制度改革，进一步精简审批项目。开展新一轮政府职能转变和机构改革工作。继续深化完善简政强镇事权改革，规范镇级权力运行。全面推开并基本完成商事登记制度改革，推进工商注册制度便利化。深化企业投资管理体制改革。加快推进市县镇三级电子政务网络平台升级扩容，逐步将政务网络服务延伸到行政村（居）。拓展公共资源交易范围，建设统一的公共资源网上交易平台。

推进财政体制改革。加强财政预算管理，积极推进预决算信息公开，市本级实行零基预算。盘活财政存量，下大力气清理部门历史沉淀资金。优化财政增量，加强财政性资金使用方向和绩效评估，财政性投资项目严格按核定的投资规模建设。集中有限财力办大事，专项资金50%以上用于重点项目（事项）。提高非税收入政府统筹比例。严格控制“三公”经费支出，压缩一般性支出。保障重点项目前期经费和启动资金。

创新融资模式。完善市属国有企业现代企业管理制度，强化资本运作，提高市场化水平。支持交投集团等企业参与政府投资基建项目建设。鼓励茂名港集团、高新开发集团、新城控股集团等企业引入战略投资者。支持有条件经济功能区探索建设一定比例商住项目，解决土地开发和项目投入问题。加强风险防范，充分评估政府性融资项目风险，认真考虑还款来源和还款渠道，努力实现债务平衡。加快推进企业上市工作，争取1家以上企业在“新主板”上市。

提升经济开放度。着力推进园区招商、产业招商和反哺工程，力争在农业产业化、临港产业和大型城市综合体引资等方面取得新突破。抓好外贸转型升级示范基地和服务平台建设，建立重大外资项目分级协调机制。抓紧市进出境货运车辆检查场后续建设，年内建成营运。支持推动民营经济加快发展，密切与外地茂名商会沟通联系。加强矿产资源整合，大力引进有实力投资者。进一步改善营商环境，吸引大型企业功能性机构和区域总部集聚茂名。

（五）着力推进生态环境保护，加强生态文明建设。

强化生态敏感区环境治理。年内完成全市生态控制线划定工作。严格落实高州水库水源和生态保护各项决定事项，突出抓好库区污水处理厂、垃圾站场、生态公益林等工程项目，下大力气清理库区养殖场。加大罗坑水库和鉴江、罗江、袂花江、东干渠、工业河、儒洞河等饮用水源保护力度，建成河东水厂第二条输水管线，强化饮水安全保障。

加强生态屏障和环保设施建设。扎实推进绿化茂名大行动各项工作，突出抓好国省道沿线绿化工程。积极开展南粤水更清行动，建成河西城区生活污水处理厂、茂南污水处理厂，开工建设茂港区、滨海新区生活污水处理厂，全面铺开15个中心镇污水处理设施建设，提高城镇污水处理率。积极推进市环保产业循环经济示范中心项目建设。

加大环境污染治理力度。继续抓好白沙河、小东江污染治理。坚决关闭小炼油厂、小砖厂、小皮革厂等污染企业。加大环保执法力度，对涉嫌环境污染犯罪的，一律移送司法机关依法追究刑事责任。积极开展保护市区空气质量专项行动。抓好陆源入海排污和近岸海域海水监测。大力加强海岸线保护管理。加快茂名石化炼油厂卫生防护距离内居民搬迁安置工作。

加强节能减排工作。全面推进清洁生产，加大重点行业污染治理力度。促进循环经济发展，支持园区申报省循环经济示范区。严把环保准入关，重点完成水泥、皮革、造纸等行业淘汰落后产能计划。

（六）着力推进以民生为重点的社会事业，共建共享和谐茂名。

推进充分就业和全民社保工作。巩固推进就业“双百”工程，全年实现城镇新增就业6万人，城镇失业人员再就业1.7万人。培训农村劳动力3.2万人，转移就业8万人。巩固社保扩面征缴成果，实施城乡居民大病保险，提高企业退休人员基本养老金水平。加大公共租赁住房等保障性住房供给，抓好棚户区改造。做好农村低收入住房困难户住房改造和“两不具备”贫困村庄移民搬迁安置工作。抓好底线民生，提升低保户、五保户、残疾人、孤儿保障水平，提高城乡居民养老保险基础养老金和医保筹资标准。积极发展养老服务事业，年内全市各类养老床位达到12000张。

大力提高科教文卫体发展水平。实施创新驱动战略，建设一批科技创新服务平台，推动国家知识产权试点城市创建工作。创建41个教育强镇（街），实现教育强镇全覆盖。切实加强职业教育，推动茂名卫生学校创办卫生职业学院。加快推进公立医院综合改革工作。提高乡村医生队伍医疗技术水平，完善全市镇村卫生服务一体化管理工作。鼓励引导社会资源投资办医，发展民营医院。切实加强人口计生工作。努力推动文化事业繁荣发展，打造地方文化活动品牌，开展文化“三下乡”活动。加强城市社区体育公园和乡镇农民健身工程建设，广泛开展群众体育活动。办好市第十二届运动会。切实做好第三次全国经济普查。

切实推进平安茂名建设。推进治安重点地区和突出治安问题集中整治，深化“以民促安”打防体系建设，推进“全城响应和快速反应”系统向郊区乡镇延伸，推进安保点和“平安家居”社会视频规范化建设。规范互联网建设管理，重视网络舆情。广泛开展社会矛盾排查化解，改革涉法涉诉信访工作，推行信访终结制度，推行重大决策社会稳定风险评估。完善突发事件应急处置机制。落实安全生产“党政同责、一岗双责、齐抓共管”要求，加强安全生产基层基础建设，突出抓好油气管道、石油库、危化品运输、道路交通等行业领域专项整治，坚决遏制重特大事故。强化食品药品安全重点整治。做好国防动员与教育、双拥、优抚安置工作，巩固军政军民团结。

各位代表！加强政府自身建设，是全面完成今年各项目标任务的重要保证。市政府将按照为民务实清廉的要求，始终保持奋发有为的精神状态，深化改革、开拓创新、真抓实干，努力建设法治政府和服务型政府。一是加强民主法治建设。认真落实人大及其常委会各项会议决定，坚持向人大及其常委会报告工作，自觉接受监督。主动加强同人民政协的联系并向其通报工作。认真办理人大代表建议和政协委员提案。广泛听取各民主党派、工商联、无党派人士和各人民团体意见。巩固和扩大基层民主。加强政府法制工作，完善规范性文件、重大行政决策合法性审查机制，加强行政复议、行政应诉等工作。二是切实改进工作作风。落实中央“八项规定”和我市实施办法，扎实开展群众路线教育实践活动，全力打好解决“四风”突出问题攻坚战，建立密切联系群众、改进作风常态化制度。坚持深入现场、深入项目、深入群众，更多地下沉一级开展调查研究和督办工作。发扬“钉钉子”精神，牢牢盯住重点工作和重点项目，反复督促检查，实打实把一件件事做好。三是创新工作方式方法。突出重点抓工作，继续围绕交通基础设施、产业园区建设、中心城区扩容提质“三大抓手”，依托博贺湾经济区、高新区、水东湾新城“三大平台”，着力突破土地、资金、人才、体制瓶颈。加强和创新督查方式，按照省的部署，启动我市督查平台建设，确保各项决策部署落到实处。四是狠抓勤政廉政建设。严格落实《党政机关厉行节约反对浪费条例》、《党政机关国内公务接待管理规定》等要求，最大限度压缩行政经费支出。推进行政审批、“三公”经费等重点领域信息公开和政务服务体系建设。强化行政监察和审计监督，加强领导干部经济责任审计和重大事项报告核查。加强公务员教育管理和能力建设，努力打造政治坚定、能力过硬、清正廉洁、作风优良、奋发有为的高素质公务员队伍。

各位代表，展望新的一年，我们满怀信心！让我们团结一致，在市委的正确领导下，全面贯彻落实党的十八届三中全会精神，解放思想，锐意进取，奋力开创茂名经济社会发展新局面！
